# Association between Psychological Suffering and Suicidal Thinking in Patients with Urologic Cancer Using Real-World Data

**DOI:** 10.3390/jcm11247336

**Published:** 2022-12-09

**Authors:** Kounseok Lee, Gyoohwan Jung, Na Yeon Choi, Sunhae Kim, Jung Ki Jo

**Affiliations:** 1 Department of Psychiatry, College of Medicine, Hanyang University, Seoul 04763, Republic of Korea; 2 Department of Urology, Seoul National University Bundang Hospital, Seongnam 13620, Republic of Korea; 3 Biostatistical Consulting and Research Laboratory, Medical Research Collaborating Center, Hanyang University, Seoul 04763, Republic of Korea; 4 Department of Urology, College of Medicine, Hanyang University, Seoul 04763, Republic of Korea

**Keywords:** cancer, suicide, depression, urology

## Abstract

Cancer is a leading cause of death in Korea, and depression and suicide are major psychiatric problems in cancer patients. This study aimed to explore the correlation between anxiety, depression, social support, cancer state, and suicidality among urologic cancer patients. Sixty patients with urologic cancer were admitted to a university hospital between October 2019 and February 2020. The patients were evaluated using the Patient Health Questionnaire-9, Generalized Anxiety Disorder 7-item scale, Suicidality module of the Mini International Neuropsychiatric Interview (MINI), and the Lubben Social Network scales (LSNS). To determine which psychological or demographic factors affected suicide risk, Fisher’s exact test, Wilcoxon rank-sum test, regression, and logistic regression were conducted. It was found that the greater the depressive symptoms, the higher the suicidal risk (OR = 1.32, 95% CI = 1.08–1.61). Furthermore, anxiety symptoms and the duration of cancer after diagnosis significantly increased depressive symptoms (*p* = 0.032). Clinicians should be able to identify the risk factors for suicide in patients with cancer, one of which is depression. To assess the risk of suicide, we must evaluate not only depressive symptoms but also the related anxiety and duration of the disease. Prevention and intervention efforts are needed to improve depressive moods and anxiety after cancer diagnosis.

## 1. Introduction

According to health statistics published by the Organization for Economic Co-operation and Development (OECD) in 2016, the age-standardized suicide mortality rate in Korea is 43.3 for men and 16.8 for women per 100,000 people. This ranks Korea first among member countries, far exceeding the OECD average of 18.9 and 5.5 for men and women, respectively [1]. According to a 2005 study, the socioeconomic cost of suicide was estimated to reach a maximum of 4 trillion won [2]. In addition, family members have been reported four times more likely to seek medical help after a relative commits suicide, indicating that it has significant negative physical and psychological effects on family members [1,2].

According to an epidemiological survey on mental illness, 15.6% of adults have experienced serious suicidal thoughts at least once in their lifetime, whereas 3.3% and 3.2% planned and attempted suicide, respectively [3]. Moreover, although 27.6% of the total population experienced mental illness at least once in their lifetime, only 15.3% received counseling or treatment from a psychiatrist or other mental health professional, and 85% were found to have never used psychiatric services [3].

Many studies have reported that those who attempt suicide only consider it for a short period of time, up to an hour before the attempt [4,5]. Even when fatal suicide methods are used, many suicidal deaths are impulsive rather than planned [6]. Moreover, suicide is a result of a combination of factors, such as social, biological, personal, and mental health factors, as well as stress-causing life events in a continued line of suicidal beliefs, motivations, and actions. Thus, it is necessary to comprehensively understand various factors underpinning this phenomenon [4,5,6].

One study reported that 7.8% of cancer patients considered suicide in order to escape the fear of cancer progression, stress, and suffering [7]. Severe pain, a high number of hospitalizations, metastasis to other organs [8], repeated recurrence [9], loss of autonomy and control over the situation, and feelings of helplessness and despair (Lefetz and Reich, 2006) led to this increased risk [9,10].

Although suicidal thoughts do not necessarily result in death due to suicide, they are an important predictor of suicide attempts, regardless of the severity of suicidal thoughts. Therefore, this study aims to analyze the presence and absence of suicidal events as surrogate markers of suicide in patients with cancer. Moreover, this study aimed to develop clinical support for cancer patient care by analyzing related factors.

## 2. Materials and Methods

### 2.1. Subjects

After Institutional Review Board (IRB) approval, patients who were diagnosed with or treated for cancer and visited the tertiary urology department from October 2019 to March 2020 were selected for participation. The emotional state of patients undergoing treatment for urinary tumors was assessed, and suicidal thoughts and depression were analyzed. A survey was also conducted to collect basic information such as age, sex, and other underlying conditions. The type of cancer, its recurrence, its progression, and its association with depression were then analyzed.

### 2.2. Measures

#### 2.2.1. The Patient Health Questionnaire

The patient health questionnaire (PHQ-9) consists of nine questions that were developed based on the nine-item diagnostic criteria of a major depressive episode from the Diagnostic and Statistical Manual of Mental Disorders, 4th Edition (DSM-IV), which are used to assess depressive symptoms [11] in the preceding two weeks. The total score ranges from 0 to 27, whereby the higher the score, the more severe the depression. The validity and reliability of the Korean version of the PHQ-9 were confirmed in a previous study [12].

#### 2.2.2. Generalized Anxiety Disorder

The Generalized Anxiety Disorder 7-item (GAD-7) is a self-reported questionnaire that was developed by Spitzer in 2006 to screen for GAD and assess its severity in clinical practice and research [13]. This study used it to measure participants’ anxiety levels. The total score ranges from 0 to 27, whereby the higher the score, the higher the anxiety level.

#### 2.2.3. Lubben Social Network Scales

The social support network was assessed using the Lubben Social Network Scales (LSNS), which is a 10-item scale measuring five aspects of social networks: family networks, friend networks, helping others, confidant relationships, and living arrangements [14]. In our study, we used a version that was translated into Korean and had been verified for reliability and validity [15].

#### 2.2.4. Suicide Risk Assessment

The Mini-International Neuropsychiatric Interview (MINI) is a simple and structured interview tool developed in 1998 for the diagnosis of major Axis I psychiatric diseases of DSM-IV and ICD-10 (International Classification of Diseases 10th Edition). In this study, six questions related to suicide were used in the self-report form.

### 2.3. Statistical Analysis

Fisher’s exact test, Wilcoxon rank-sum test, regression, and logistic regression analyses were performed using SAS version 9.4 (SAS Institute Inc., Cary, NC, USA) to analyze basic information according to the depression group, the effects of several variables (by setting the dependent variable as the suicidal thoughts group), and the effects of depression (by setting the dependent variable as the total depression score).

## 3. Results

A total of 60 urinary cancer patients who completed all the questionnaires were included in the analysis. Table 1 shows the results of the analysis of differences in basic information (age, height, etc.) across the different depression groups. Depression was classified as mild, moderate, or severe based on the total PHQ-9 score. As none of the participants had severe depression, they were divided into mild and moderate depression groups. There were no differences between the two groups except for anxiety.

The suicidal thoughts group was set as the dependent variable, and the effects of variables such as age, sex, and total depression score on the suicidal thoughts group were then assessed (Table 2). In both univariate and multivariate analyses of suicidal thoughts, there were no significant results for the variables.

The total depression score was set as a dependent variable to evaluate the effects of suicidal thoughts, anxiety, and the duration of cancer after diagnosis (Table 3). The duration of cancer after diagnosis was divided into 12, 24, and 36 months to compare the multivariate models. However, the differences were not statistically significant.

In the LSNS, the scores of questions one, two, and three, indicating family bonds, were set as family. The results of the comparative analysis of families with depression, including the duration of cancer after diagnosis (months) and interactions between the two, showed that suicidal thoughts, anxiety, and duration of cancer after diagnosis were associated with depression (Table 4, Family: *p* = 0.006, Anxiety: *p* < 0.001, period after cancer diagnosis: *p* = 0.032). Moreover, when the effects of family bonds and interactions on the duration of cancer after diagnosis were assessed, it was found that interaction had an effect on depression (Interaction: *p* = 0.03).

In the univariate analysis of depression, the type of cancer, recurrence, and disease progression were not significant; however, significant outcomes were observed in the presence or absence of recurrence in the multivariate analysis (*p*-value = 0.038) (Table 5).

## 4. Discussion

In this study, psychological problems, which are often overlooked in the treatment of cancer patients in clinical settings, were analyzed and reported. In particular, the degree of depression, including suicidal thoughts, was reported; the effort of clinicians and the active support of family, including the spouse, were found to be influencing factors.

Suicide is a series of processes that start with suicidal thoughts, leading to suicide attempts and eventual completion. Suicidal thoughts are known to be a risk factor for suicide attempts (Kessler et al., 1999), and suicide planning is also a risk factor that is likely to lead to suicidal thoughts [16,17].

Moreover, cancer diagnosis is often equated with death, which can cause a physical and psychological crisis in the patient and their family [18]. When patients are diagnosed with cancer or learn about a relapse during treatment, they show emotional reactions such as shock, distrust, anxiety, depression, confusion, and fear [18,19].

As observed in this study, the duration of cancer after diagnosis affects depression, indicating that it is associated with suicidal death. This also suggests that patients thought of death differently than before or during their cancer diagnosis.

It has been reported that more than 90% of suicides are preceded by psychiatric diseases [20]. Among them, the suicide rate is very high for diseases such as substance use disorders, anxiety disorders, personality disorders, and mood disorders, which is the disease that is most commonly associated with suicide. It was reported that 45% of patients with depression attempted suicide, and accounted for 15% of those who attempted suicide [21]. According to a Swedish cohort study of 39,658 people who attempted suicide, coexisting psychiatric disorders, mood disorders, and schizophrenia showed the highest risk of re-attempting suicide within one year after the initial attempt [22]. Substance use disorders also had a significant effect on suicide. It is known that approximately 40% of suicide deaths and those who attempt suicide have alcohol use problems, and the suicide rate of this group is 9.8 times higher than that of the general population [23,24]. Many previous studies have focused on suicidality within this patient group [20,21].

In the current study, anxiety disorder was associated with depression, which is, in turn, associated with suicidal thoughts. The suicidal thought rate is inevitably higher in cancer patients who use various drugs, and a special multidisciplinary system is required to care for such patients.

Although suicide risk in clinical settings can be assessed through the evaluation of impulsiveness and depression in addition to personality assessment, these various evaluation tools are often used only in psychiatry. However, in actual clinical settings, it is necessary to use such tools for bladder cancer patients who have a ureter installed or for prostate cancer patients who experience complications after a prostatectomy. The frustration of disability in patients with urinary tract disorders, such as bladder cancer, their feeling of loss after organ removal, and the psychological difficulties or frustrations after surgery are often overlooked in clinical practice. Moreover, problems such as urinary incontinence or erectile dysfunction, which can occur after prostatectomy for prostate cancer, can cause extreme incidents. Due to these different feelings of loss or frustration in the clinic, patients rarely mention suicide before medical appointments. There is a lack of multidisciplinary treatment systems for these patients, and it is necessary to establish a patient care system to address this.

Anxiety and despair might be the result of oncological treatment-related functional effects. The increase in asymptomatic prostate cancer diagnosis (such as over-treatment) and reports of subsequent functional side effects can be said to reflect this [25].

It was reported that suicidal thoughts increased when there was no spouse who was the primary source of emotional and instrumental support for the patient [26]. In this study, bonds with family members, including spouses, were analyzed. A tendency similar to that observed in previous studies was also observed herein, and it seemed to be related to depression. Filiberti et al. reported that factors affecting suicide vary and can include mental distress during the treatment process, uncontrolled pain, and other physical discomforts. However, it was also reported that suicide can be prevented if physicians provide adequate psychosocial support along with appropriate medications [27]. It is also necessary to develop a multidisciplinary system that can increase the sense of bonding with the family, even in patients with urinary system cancer [26,27].

Furthermore, it was reported that fear of transmission during the coronavirus pandemic had a positive correlation with the stress level of urological patients [28]. Occasionally, treatment methods are limited because of concerns regarding infection, and measures have been reported [29]. This study was conducted during the SARS-CoV-2 era and was able to show a correlation between anxiety and depression in urological cancers, one of the major problems in the urology sector that can affect outcomes.

In prostate cancer, customized treatments such as anti-cancer gene therapy using suicide genes and adenovirus vectors have been proposed [30], and multidisciplinary treatments to reduce anxiety and depression through these customized treatments should be considered.

This study has several limitations. First, there were limitations in fully trusting the patients’ responses, as they were self-reported. Second, there was no detailed information on the fatality, method, and frequency of suicide. In addition, there was no information regarding the psychiatric treatment history of the participants. Third, the survey was conducted at only one hospital. Thus, it is difficult to generalize the results across all patients. Fourth, this is a cross-sectional study. Therefore, it was not possible to clearly define the causal relationship between the related pathological factors and suicide risk. Future studies ought to comprehensively evaluate environmental and emotional factors and conduct continuous follow-ups of suicide risk. Although there are limitations to this study, the emotional state of patients diagnosed with and being treated for urinary tumors was assessed, and the basis for establishing an improved treatment system and emotional coping was presented.

## 5. Conclusions

In this study, depression, suicidal thoughts, and related factors in patients diagnosed with urinary system cancer, including bladder cancer patients with urinary fistulas, were analyzed. It was observed that the development of a customized multidisciplinary system, including professional counseling from the Department of Psychiatry, was required to induce family bonding according to the patient’s characteristics. Moreover, physicians need to use the system so that the patient is not only treated for cancer but also for their overall well-being.

## Figures and Tables

**Table 1:** General patient information.

Variables	No (*n* = 49)	Yes (*n* = 11)	*p*-Value
Age (years)	72.0 ± 9.0	67.5 ± 8.4	0.102
Sex			
Male	45 (91.8)	8 (72.7)	0.108
Female	4 (8.2)	3 (27.3)	
B total	2.3 ± 2.9	5.5 ± 3.7	0.006
Anxiety (yes)	5 (10.2)	3 (27.3)	0.154
Satisfaction			
Satisfied	26 (53.1)	4 (36.4)	0.441
Neutrality	3 (6.1)	0 (0.0)	
Dissatisfied	20 (40.8)	7 (63.6)	
HTN (yes)	33 (67.4)	4 (36.4)	0.086
DM (yes)	12 (24.5)	2 (18.2)	1.000
Other disease (yes)	28 (57.1)	4 (36.4)	0.212
Months	52.9 ± 62.8	27.8 ± 36.0	0.069
Current status			
Local	36 (73.5)	7 (63.6)	0.121
Locally advanced	10 (20.4)	1 (9.1)	
Advanced	3 (6.1)	3 (27.3)	
Cancer type			
Prostate cancer	26 (53.1)	3 (27.3)	0.238
Bladder cancer	14 (28.6)	4 (36.3)	
Kidney cancer	4 (8.1)	2 (18.2)	
Ureteral renal cancer	3 (6.1)	2 (18.2)	
Etc.	2 (4.1)	0 (0.0)	
Progression at diagnosis			
Local	37 (75.5)	7 (63.6)	0.178
Locally advanced	8 (16.3)	1 (9.1)	
Advanced	4 (8.2)	3 (27.3)	
Recurrence			
Yes	9 (18.4)	2 (18.2)	1.000
No	40 (81.6)	9 (81.8)	

Wilcoxon rank-sum test.

**Table 2:** Logistic regression of suicidal ideation.

Variables	Univariate		Multivariate *	
	OR (95% CI)	*p*-Value	OR (95% CI)	*p*-Value
Age (years)	0.95 (0.88–1.02)	0.141		
Sex (male)	0.24 (0.04–1.27)	0.092		
B total	1.32 (1.08–1.61)	0.006	1.23 (0.82–1.84)	0.313
Months	0.99 (0.97–1.01)	0.228		
Months group (≥12)	0.28 (0.07–1.11)	0.070	0.16 (0.01–1.82)	0.139
Months × B total	1.00 (1.00–1.00)	0.464		
Months group × B total	1.16 (0.96–1.40)	0.120	1.15 (0.74–1.79)	0.545
Anxiety (yes)	3.30 (0.66–16.63)	0.148	0.65 (0.08–5.36)	0.692
Satisfaction				
Satisfied	Ref.			
Neutrality	<0.001 (<0.001–>999.999)	0.975		
Dissatisfied	2.28 (0.58–8.86)	0.236		
HTN (yes)	0.28 (0.07–1.09)	0.066		
DM (yes)	0.69 (0.13–3.62)	0.656		
Other disease (yes)	0.43 (0.11–1.66)	0.220		
Current stage				
1	Ref.			
2	0.51 (0.06–4.69)	0.555		
3	5.14 (0.86–30.91)	0.074		

* B total, months group, month group × B total, anxiety.

**Table 3:** Multivariate Regression analysis for Depression.

Variables	Estimate	Standard Error	t	*p*-Value
Suicidal ideation (yes)	2.245	1.027	2.19	0.033
Anxiety (yes)	4.834	1.233	3.92	<0.001
Months1 (>12)	0.115	0.802	0.14	0.887
Suicidal ideation × Anxiety	0.814	2.156	0.38	0.707

**Table 4:** Regression analysis of depression.

Variables	Univariate				Multivariate			
	Estimate	Standard Error	t	*p*-Value	Estimate	Standard Error	t	*p*-Value
Anxiety (yes)	5.596	1.013	5.53	<0.0001	5.661	1.015	5.58	<0.0001
Family	−0.027	0.142	−0.19	0.847	0.266	0.169	1.57	0.123
Months	−0.003	0.007	−0.41	0.683	0.054	0.029	1.87	0.067
Family × Months	−0.001	0.001	−0.72	0.474	−0.006	0.003	−1.99	0.052

**Table 5:** Regression analysis of depression.

Variables	Univariate				Multivariate			
	Estimate	Standard Error	t	*p*-Value	Estimate	Standard Error	t	*p*-Value
Cancer type								
Prostate cancer	Ref				Ref			
Bladder cancer	0.356	0.976	0.37	0.716	0.168	0.983	0.17	0.865
Kidney cancer	1.856	1.459	1.27	0.209	2.188	1.615	1.35	0.182
Ureteral renal cancer	2.490	1.575	1.58	0.120	3.008	1.589	1.89	0.064
Etc.	2.690	2.378	1.13	0.263	−0.059	2.591	−0.02	0.982
Recurrence (yes)	1.904	1.070	1.78	0.081	2.700	1.265	2.13	0.038
Progression at diagnosis								
Local	Ref				Ref			
Locally advanced	1.523	1.187	1.28	0.205	1.382	1.206	1.15	0.257
Advanced	1.666	1.320	1.26	0.212	0.619	1.583	0.39	0.697
Progression								
No	Ref.				Ref.			
Yes	−2.948	3.329	−0.89	0.380	−4.368	3.440	−1.27	0.210

## Data Availability

The data that support the findings of this study are available upon request from the corresponding author.
